# Intake of partially defatted Brazil nut flour reduces serum cholesterol in hypercholesterolemic patients- a randomized controlled trial

**DOI:** 10.1186/s12937-015-0036-x

**Published:** 2015-06-16

**Authors:** Roberta F Carvalho, Grazielle V B Huguenin, Ronir R Luiz, Annie S B Moreira, Glaucia M M Oliveira, Glorimar Rosa

**Affiliations:** 1 Post Graduate Program, Federal Universityof Rio de Janeiro, Professor Rodolpho Paulo Rocco St, 225 Rio de Janeiro, Brazil; 2 Institute of Public Health Studies, Federal Universityof Rio de Janeiro, Jorge Machado Moreira Square, 100 Rio de Janeiro, Brazil; 3 ClinicofAtherosclerosisand Cardiovascular DiseasePrevention, National Cardiology Institute, Laranjeiras St, 374 Rio de Janeiro, Brazil; 4 Nutrition and Dietetic Department, Josué de Castro Institute of Nutrition, Federal Universityof Rio de Janeiro, 373, 2nd floor, block J, Carlos Chagas FilhoAv, Ilha do Governador, 21941-902 Rio de Janeiro, Brazil

**Keywords:** Brazil nut, Selenium, Serum lipids, Thyroid hormones, Dyslipidemia

## Abstract

**Objective:**

Thyroid hormones can lower levels of atherogenic lipoproteins, and selenium is important in thyroid hormone homeostasis. We aimed to investigate the effects of a healthy diet associated with the Brazil nut (*Bertholletia excelsa*) in dyslipidemic and hypertensive patients.

**Methods:**

This study was a randomized, placebo-controlled, double-blind trial. Seventy-seven dyslipidemic and hypertensive patients already receiving lipid-lowering drugs received either a dietary treatment associated with partially defatted Brazil nut flour (13 g/day providing 227,5 μg of selenium/day),or with dyed cassava flour as a placebo. All patients received a personalized dietary guideline with nutritional recommendations for dyslipidemia and hypertension and were followed for 90 days.

**Results:**

The Brazil nut group showed reductions in total cholesterol (−20.5 ± 61.2 mg/dL, *P* = 0.02), non HDL-cholesterol (−19.5 ± 61.2 mg/dL, *P* = 0.02) and Apo A-1 (−10.2 ± 26.7 mg/dL, *P* = 0.03) without significant alterations in the Apo B/Apo A-1 ratio. The placebo group showed a reduction in FT_3_ levels (−0.1 ± 0.4, *P* = 0.03) and increased Lp(a) levels (5.9 ± 18.0 mg/dL, *P* = 0.02). There were no statistical differences in blood pressure and serum lipids between Brazil nut and placebo group.

**Conclusions:**

Supplementation with Brazil nuts seems to favor the maintenance of FT_3_ levels and contributes to lipemia reduction in hypercholesterolemic and euthyroid patients.

**Trial registration:**

ClinicalTrials.gov Identifier NCT01990391

## Introduction

Atherosclerotic cardiovascular disease is has a high worldwide prevalence and mortality [1]. Hyperlipidemia increases the incidence and risk of this disease, as confirmed by several large-population studies [1, 2].

It has been described that thyroid hormones and thyromimetic drugs has LDL cholesterol (C) lowering effec [3−7] and also reduce levels of other atherogenic lipoproteins such as triglycerides [5, 7], apolipoprotein B (apo B) [6] and lipoprotein (a) (Lp(a)) [5−7], although they also decrease levels of non-atherogenic lipoproteins, such as apolipoprotein A-1 (apo A-1) [3]. These effects may be caused by stimulation of LDL receptors, which increases hepatic clearance and cholesterol elimination, and presumably promotes reverse cholesterol transport, even in individuals without subclinical hypothyroidism [6, 8, 9].

Selenium is an essential micronutrient for the metabolism of thyroid hormones, playing a major role in thyroxine conversion to the more active metabolic form triiodothyronine because the activity of some deiodinases is dependent on selenium [10, 11]. The thyroid gland contains the highest amount of selenium [10], and selenoprotein glutathione peroxidase can protect the thyroid gland from the oxidative damage produced during thyroid hormone synthesis [12]. Selenium also plays an important antioxidant role in cardiovascular disease and studies have shown that serum and urinary levels of selenium are lower in patients who suffered acute myocardial infarcts or ischemic cardiomyopathy [13].

The Brazil nut (*Bertholletia excelsa*, family Lecythidaceae) originates from the Amazon region and is the richest known food source of selenium [14]. Most selenium content in this nut is present in protein fractions;therefore, the cake protein has the highest amount of selenium presented as selenomethionine and selenocysteine [15]. The high concentration of selenium in the Brazil nut may improve serum lipoprotein, due to its stimulation of the metabolically active form of thyroid hormones.

The present study investigated the effects of a healthy diet associated with defatted Brazil nut flour on thyroid hormones and in serum lipoproteins in dyslipidemic and hypertensive patients.

## Patients and methods

### Ethical considerations

The Ethics Committee for Clinical Research of the National Cardiology Institute approved this study (protocol number 316/2011) in Rio de Janeiro, Brazil. This study was also registered at ClinicalTrials.gov (NCT01990391). All patients were informed about the procedures and gave their written informed consent.

### Study patients

Eighty-nine dyslipidemic and hypertensive patients (49 men and 40 women) were recruitedat the National Institute of Cardiology at the Dyslipidemia and Atherosclerosis Outpatient Clinic. Eligible patients were males and females aged 40 to 80 years old, with referred diagnoses of dyslipidemia and hypertension who were taking medication for both conditions for at least 3 months before being included in the study, and were diagnosed with euthyroidism. Euthyrodism was defined as thyroid stimulating hormone (TSH) (reference range, 0.45 – 4.50 μUI/mL) and free thyroxine (FT4) (reference range, 0.70 – 1.48 ng/dL) within the normal reference range [16].

Patients with history of thyroid disease, thyroid medication use, chronic renal failure with glomerular filtration rate < 60 mL/min/1.73 m^2^ [17] were excluded. As well as those currently or previously having ingested supplements containing > 20 μg Se/day, or presenting excessive consumption of Brazil nuts in the past year, having plasma selenium levels above 125 μg/L [18], being current smokers [19], and having been in a rigorous exercise/weight-reduction program within the 3 months before entering the study.

### Experimental design

This study was a 90-day randomized, placebo-controlled, double-blind clinical trial. Eligible patients were randomly assigned to one of two groups: diet + partially defatted Brazil nut flour (*n* = 35) or diet + placebo (artificially flavored dyed cassava flour) (*n* = 42).

Patients were randomized using the method of computer generated random list restricted in blocking of 10 patients and sequentially numbered labels were inserted in sealed containers with Brazil nut or placebo. The researcher that generated the random allocation sequence was not the same that enrolled participants and assigned participants to interventions.

Patients were assessed before and during the 90-day trial, with monthly blood test to measure thyroid hormones, lipid profile, and plasma selenium levels. In addition, food intake was analyzed through patients’ dietary anamnesis whereas anthropometric data were evaluated through measures of waist circumference (WC) and body weight and height, for calculation of body mass index (BMI). At each visit, patients were given a sealed opaque flask containing either 450 g of Brazil nut flour or placebo, and standard measuring spoons. Plasma selenium levels were used as markers of compliance to Brazil nut consumption.

Self-reported questionnaires were used to determine physical activity [20], dietary habits, and medications. Physical exercise was taken into account when patients exercised at least once a week, and was reported as Metabolic Equivalent of Task (MET), expressed in kcal/day [21]. Patients were asked to maintain their normal amount of physical activity and dose of lipid-lowering medication. This was verified by analyzing questionnaires filled out by patients and by examining the medical record containing prescription information of each patient visit.

### Nutritional intervention

Patients received either 13 g/day of partially defatted Brazil nut flour providing 64.4 kcal, 0.17 g carbohydrate, 3.4 g protein, 5.56 g total fat, 2.58 g dietary fiber and 227.5 μg of selenium/day (Ouro Verde Amazônia® – Mato Grosso, Brazil) or 11 g/day of artificially flavored dyed cassava flour (Mane of Brazil Industry and Commerce Ltda – Rio de Janeiro, Brazil). The nutritional composition 11 g of placebo is: 36.5 kcal, 8.92 g carbohydrate, 0.12 g protein, 0.03 g total fat, 0.65 g dietary fiber [22] and 0.07 μg selenium [23]. The volume of Brazil nut and placebo was different because of the total volume of the opaque flask used to store the supplements. Since placebo was less dense, it requires a higher volume and more flasks, so it could difficult the double blinding of the study.

Partially defatted granulated Brazil nut was used in this study rather than the Brazil nut kernel because the granulated Brazil nut has a higher Se content than the Brazil nut kernel (227,5 μg vs. 249,21 μg [22, 23], respectively) and they have similar centesimal compositions except for less total fat and fewer calories. In addition, granulated Brazil nut allowed for blinding the study and is already commercialized.

The amount of selenium present in the Brazil nut flour was measured by flame atomic absorption spectrometry. The energy value of Brazil nut was 59 kcal/day while the placebo group was 46.8 kcal/day.

The dietary plan was prescribed in accordance to the volunteers’ dietary habits and nutritional recommendations according to guidelines for dyslipidemia and hypertension [24, 25]. Participants also received a weekly menu and healthy recipes. Dietary compliance was verified monthly at the check-up via 24 h diet reminders that were analyzed with the Food Processor software, version 12 (EshaResearch, Salem, USA, 1984).

### Blood collection and biochemical evaluation

Blood samples were collected following 12 h of overnight fasting and analyzed at the Clinical Analyses Laboratory of National Cardiology Institute (Rio de Janeiro, Brazil) using an automated method (ARCHITECT *ci*8200, Abbott ARCHITECT®, Abbott Park, IL, USA) and commercial kits (Abbott ARCHITECT *c*8000®, Abbott Park, IL, USA).

Serum TSH and thyroid hormones free triiodothyrodine (FT3) and free thyroxine (FT4) were measured by immunochemiluminescence of microparticle [26, 27] with analytical sensitivity of 1 pg/mL for FT3, 0.4 ng/dL for FT4, and 0.0025 μUI/mL for TSH.

Serum concentrations of triglycerides, total cholesterol, and HDL-c were assayed by enzymatic-colorimetric methods: glycerol phosphate oxidase/peroxidase [28], cholesterol oxidase/peroxidase [29], and direct detergent [30], respectively. Non-HDL cholesterol was calculated as follows: total cholesterol – HDL-c [31]. LDL-c values were obtained according to the Friedewald formula [32]. Serum levels of Apo A-1 and Apo B were measured with immunoturbidimetry [33]. The Apo B / Apo A-1 ratio was obtained using the conventional values. To determine plasma Lp(a) levels, collecting was carried out with tubes containing ethylenediaminetetraacetic acid (EDTA), and analyses were conducted by particle-enhanced turbidimetric immunoassay [34].

Plasma selenium was analyzed in samples collected in NH Trace Element tubes with sodium heparin (VACUETTE®) and were maintained at −70 °C until they were analyzed with atomic absorption spectrometry using an inductively coupled plasma mass spectrometer (NexION™ 300 ICP-MS, PerkinElmer, Massachusetts, USA) [35]. This analysis was conducted at the LABSPECTRO Laboratory of the Pontifical Catholic University (Rio de Janeiro, Brazil).

### Statistical analyses

The estimated sample size was based on detection of a significant mean increase in T3 levels of 0.07 ± 0.2 nmol/l, with selenium supplementation at a dosage of 200 μg/d during three months, as one of the objectives of this study. It was used a two-sided type I error of less than 5 %. Thus, 70 patients was required for a statistical power of 80 %.

All statistical analyses were conducted using the *Statistical Package Social Sciences* (SPSS) software, version 16. Results were reported as mean ± SD. Data normality was tested by *Shapiro-Wilk W* test and the chi-square test to assess the differences in the qualitative variables between groups. To evaluate the effect of Brazil nut consumption on the investigated variables, we used the *Mann-Whitney U* test to analyze between-group differences and a *Wilcoxon Signed Ranks* test to assess changes in each group for non-normal data. Finally, for normal data, the Independent-Simple Student *t*-Test was used to compare means between groupsand the Paired-Simple Student *t*-Test was used to assess differences in each group throughout the study. Significant differences were assumed at *P* < 0.05.

## Results

**Fig. 1:**
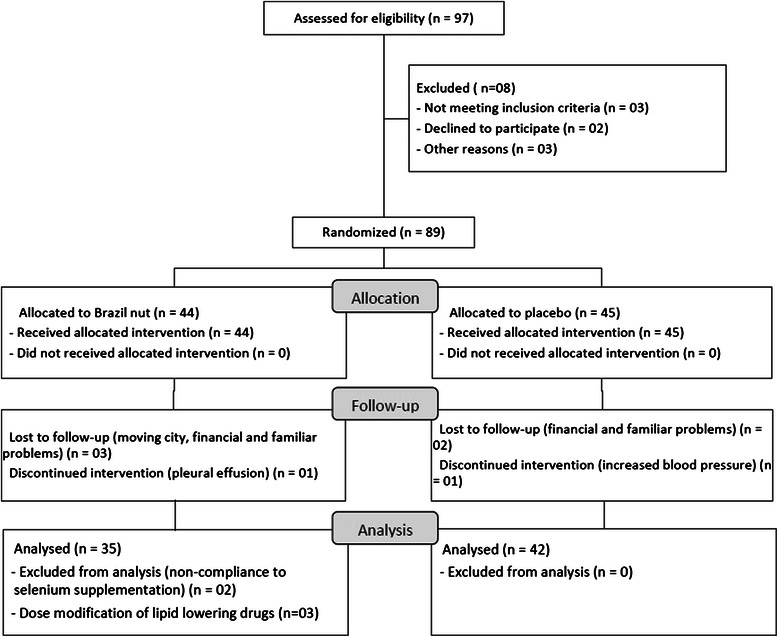
Flowchart of participants in the randomized clinical trial

Of the 89 patients randomized, 12 did not complete the study (Fig. 1). No adverse effects were reported by any of the participants. One patient discontinued consuming Brazil nut due to pleural effusion, but this condition was unrelated to the supplementation, as this patient had a history of coronary artery disease. Seventy-seven patients completed the study; 35 in the diet + Brazil nut group and 42 in the diet + placebo group. The patients who did not complete the study had the same investigated characteristics as those who completed it (Table 1).

**Table 1:** Baseline characteristics of the study and dropout patients

Characteristic	Concluded study (*n* = 77)	Drop out (*n* = 12)	*P value*
Age - years	60.05 ± 10.27	61.50 ± 9.47	NS
Male – n (%)	43 (55.8 %)	6 (50.0 %)	NS
Body mass index – kg/m^2^	29.54 ± 5.60	28.51 ± 3.38	NS
Plasma selenium - μg/L	87.57 ± 16.29	85.83 ± 18.67	NS
SBP (mmHg)	142.26 ± 28.40	136.08 ± 18.82	NS
DBP (mmHg)	82.19 ± 14.15	82.83 ± 9.47	NS
FT_3_ (pg/mL)	2.91 ± 0.41	2.93 ± 0.67	NS
FT_4_ (ng/dL)	1.20 ± 0.18	1.21 ± 0.15	NS
Total cholesterol (mg/dL)	217.88 ± 89.96	203.83 ± 56.26	NS
LDL-cholesterol (mg/dL)	130.10 ± 58.42	131.33 ± 49.22	NS
HDL-cholesterol (mg/dL)	38.83 ± 12.94	38.17 ± 9.81	NS
Triglycerides (mg/dL)	230.88 ± 224.09	194.58 ± 109.74	NS

Mean ± SD or n (%). *SBP* systolic blood pressure; *DBP* diastolic blood pressure; *FT*_*3*_ free triiodothyronine; *FT*_*4*_ free thyroxine; *LDL* low density lipoprotein; *HDL* high density lipoprotein

For quantitative variables, we used a Student’s *t* test for independent samples (selenium and age) or a Mann–Whitney *U* test for variables with normal and non-normal distributions, respectively. For categorical variables, we used a Chi-squared test. There was no significant difference between groups at baseline

At baseline, the groups did not differ in terms of age, sex, BMI, diabetes mellitus diagnosis, plasma selenium levels, physical activity, and medications (Table 2). Likewise, there was no statistically significant difference between the groups in terms of daily dietary components (Table 3) and the variables studied (Table 4) at baseline.

**Table 2:** Baseline characteristics of the study patients

Characteristics	Brazil nut (*n* = 35)	Placebo (*n* = 42)
Age – years	59.6 ± 10.8	60.4 ± 9.9
Elderly – n (%)*	19 (54.3 %)	22 (52.4 %)
Male – n (%)	20 (57.1 %)	23 (54.8 %)
Diabetes – n (%)	17 (48.6 %)	15 (35.7 %)
BMI– kg/m^2^	29.9 ± 6.5	29.3 ± 4.8
Overweight / obesity – n. (%)	27 (77.1 %)	35 (83.3 %)
Plasma selenium - μg/L	88.7 ± 15.3	86.6 ± 17.2
Physical activity – n (%)†	13 (37.1 %)	14 (33.3 %)
MET (kcal/day)	23.8 ± 46.1	27.1 ± 53.8
Drug therapy – n (%)		
Lipid-lowering drugs		
Statins	31 (88.6)	35 (83.3)
Ezetimibe	18 (51.4)	15 (35.7)
Fibrate	15 (42.8)	14 (33.3)
Antihypertensive drugs		
Diuretics	25 (71.4)	26 (61.9)
ACE Inhibitors	16 (45.7)	22 (52.4)
Calcium channel blockers	14 (40.0)	15 (35.7)
Sympatholytic	31 (88.6)	35 (83.3)
Vasodilators	16 (45.7)	20 (47.6)
ARB	13 (37.1)	14 (33.3)

Mean ± SD or n (%). *BMI* Body mass index; *MET* metabolic equivalent; *ACE* angiotensin-converting enzyme; *ARB* angiotensin II receptor blockers

* Individuals older than 60

† Expressed as the percentage of individuals who responded positively to these questions (% Yes)

For quantitative variables, we used a Student’s *t* test for independent samples (selenium and age) or a Mann–Whitney *U* test for variables with normal and non-normal distributions, respectively. For categorical variables, we used a chi-squared test. There was no significant difference between groups at baseline

**Table 3:** Dietary data for the two study groups

Dietary component (intake/day)	Brazil nut (*n* = 35) Δ T_90_ – T_0_	Placebo (*n* = 42) Δ T_90_ – T_0_	*P* value intragroup^a^	*P* value intragroup^b^	*P* value intergroup^c^
Energy intake (kcal)	- 232.3 ± 153.7	- 390.1 ± 534.0	0.030*	<0.001*	NS
Lipids (%)	0.2 ± 5.4	- 0.6 ± 7.4	NS	NS	NS
Saturated FA (%)	- 0.4 ± 2.4	- 0.7 ± 3.3	NS	NS	NS
Monounsaturated FA (%)	0.3 ± 3.0	−0.6 ± 4.1	NS	NS	NS
Polyunsaturated FA (%)	0.2 ± 1.6	0.2 ± 1.3	NS	NS	NS
n-3 polyunsaturated FA (g)	- 0.1 ± 0.3	−0.1 ± 0.4	NS	NS	NS
Carbohydrates (%)	−2.4 ± 11.1	2.5 ± 12.3	NS	NS	NS
Proteins (%)	1.8 ± 9.2	1.0 ± 10.0	NS	NS	NS
Selenium (μg)	231.1 ± 35.3	−8.2 ± 44.0	<0.001*	NS	<0.001*

Mean ± SD. FA, fatty acids; NS, non-significant

* Significant differences were assumed at *P* < 0.05

^a^ Statistical differences in Brazil nut group compared to baseline

^b^ Statistical difference in placebo group compared to baseline

^c^ Statistical differences between groups compared at T90

**Table 4:** Thyroid hormones, blood pressure and serum lipoproteins at baseline and after the 90-day trial

Variables	Brazil nut (*n* = 35) Δ T_90_ – T_0_	Placebo (*n* = 42) Δ T_90_ – T_0_	*P* value intragroup^a^	*P* value intragroup^b^	*P* value intergroup^c^
FT_3_ (pg/mL)	0.1 ± 1.1	−0.1 ± 0.4	NS	0.030*	NS
FT_4_ (ng/dL)	0.1 ± 0.6	−0.1 ± 0.1	NS	NS	NS
TSH (μUI/mL)	0.2 ± 1.8	−0.2 ± 0.9	NS	NS	0.06
SBP (mmHg)	3.5 ± 18.0	−4.0 ± 30.7	NS	NS	NS
DBP (mmHg)	−1.5 ± 13.6	−5.0 ± 15.2	NS	0.020*	NS
Total cholesterol (mg/dL)	−20.5 ± 61.2	−7.4 ± 44.5	0.020*	NS	NS
LDL-cholesterol (mg/dL)	−6.4 ± 57.9	−6.5 ± 39.5	NS	NS	NS
HDL-cholesterol (mg/dL)	−1.0 ± 5.0	0.8 ± 7.1	NS	NS	NS
Triglycerides (mg/dL)	−49.6 ± 198.2	−0.05 ± 104.1	NS	NS	NS
Non-HDL cholesterol (mg/dL)	−19.5 ± 61.2	−8.2 ± 44.5	0.020*	NS	NS
Apolipoprotein A-1 (mg/dL)	−10.2 ± 26.7	−7.9 ± 27.8	0.040*	NS	NS
Apolipoprotein B (mg/dL)	−5.2 ± 25.9	−5.0 ± 23.4	NS	NS	NS
ApoB/ApoA-1 ratio	0.02 ± 0.2	−0.06 ± 0.3	NS	NS	NS
Lp(a) (mg/dl)	−0.5 ± 23.7	5.9 ± 18.0	NS	0.02*	NS

Mean ± SD. FT_3_, free triiodothyronine; FT_4_, free thyroxine; TSH, thyroid stimulating hormone; SBP, systolic blood pressure; DBP, diastolic blood pressure, ApoB, apolipoprotein B; ApoA-1, apolipoprotein A-1; Lp(a), lipoprotein (a)

^a^ Statistical differences in Brazil nut group compared to baseline

^b^ Statistical difference in placebo group compared to baseline

^c^ Statistical differences between group compared at T_90_

* Significant differences were assumed at *P* < 0.05

For normally distributed variables (FT_3_ and FT_4_), we used a Student’s *t* test for independent samples and a paired-samples *t* test to investigate the differences within and between groups, respectively. For non-normally-distributed variables, we used a Mann–Whitney *U* test and a Wilcoxon Signed Ranks test to assess the differences within and between groups, respectively

Patients kept their usual levels of physical activity during the study, thus maintaining their caloric expenditure.

There were no significant changes in BMI (Brazil nut group: Δ T_90_ – T_0_ = −0.2 ± 0.7 kg/m^2^; placebo group: Δ T_90_ – T_0_ = −0.1 ± 0.9 kg/m^2^) and WC (Brazil nut group:Δ T_90_ – T_0_ = −0.6 ± 2.5 cm; placebo group: Δ T_90_ – T_0_ = − 0.5 ± 3.3 cm) within and between groups.

Plasma selenium concentrations increased significantly in the Brazil nut group at all time-points (Δ T_90_ – T_0_ = 80.7 ± 50.2 μg/L, *P* < 0.001). In the placebo group, on the other hand, those concentrations decreased significantly at T_30_ and T_60_ (Δ T_60_ – T_0_ = − 17.2 ± 23.4 μg/L, *P* < 0.001).

**Fig. 2:**
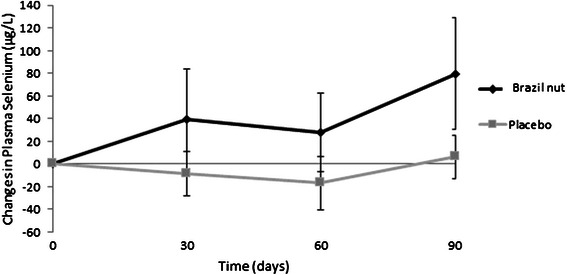
Plasma selenium levels in both groups over 90 days. Values are expressed as means and standard deviations with 95 % CI. *n* = 77. Values were measured for both groups on days 0, 30, 60 and 90. Excluding baseline levels, values differed significantly within and between groups at every time point

Selenium levels differed significantly between groups at all time-points, except at baseline (T_90_: Brazil nut = 169.5 ± 46.5 μg/L *versus* placebo = 92.7 ± 16.8 μg/L, *P* < 0.001) (Fig. 2).

Due to the nutritional recommendations, energy intake decreased significantly in both the Brazil nut (*P* = 0.03) and placebo (*P* < 0.001) groups, while selenium intake increased significantly in the Brazil nut group (*P* < 0.001) (Table 3).

Thyroid hormone profiles are presented in Table 4. There were no statistical differences in TSH and FT4 levels within and between groups, while FT3 decreased significantly only in the placebo group (*P* = 0.03) at T_90_, without alterations between groups.

**Fig. 3:**
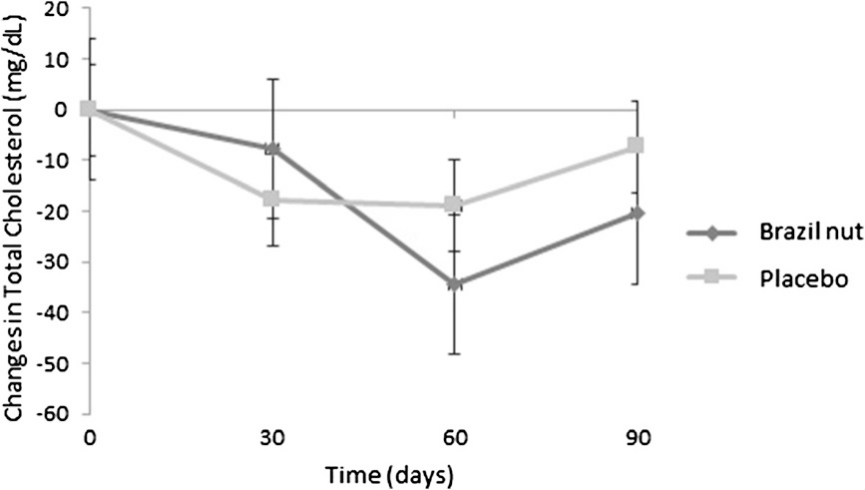
Serum total cholesterol levels in both groups over 90 days. Values are expressed as means and standard deviations with 95 % CI, *n* = 77. Values were measured for both groups on days 0, 30, 60 and 90. Values differed significantly only for the Brazil nut group at the T_90_

Serum lipoprotein levels are presented in Table 4. There were no significant differences within or between groups in LDL-c, HDL-c, triglycerides, Apo B and the Apo B / Apo A-1 ratio during the study. After 90 days, total cholesterol (*P* = 0.02), non-HDL cholesterol (*P* = 0.02) and Apo A-1 (*P* = 0.03) decreased significantly only in the Brazil nut group, however there was no difference between groups. The placebo group presented an increase in Lp(a) levels (*P* = 0.02). Figure 3 presents the changes in total cholesterol between groups during the study.

## Discussion

In this study, we analyzed the effects of a personalized balanced diet associated with defatted Brazil nut flour on the lipid levels and thyroid hormones of dyslipidemic and hypertensive patients.

Recent reports indicated that food sources naturally or artificially containing high amounts of selenium improve lipid profiles, with significant reductions in total cholesterol and non-HDL cholesterol levels [36, 37], which is supported by the present study. Moreover, Maranhão *et al.* [37] showed a significant reduction in LDL-c levels. Other authors who used selenium supplements in doses greater than 290 μg/day did not observe significant reduction in atherogenic lipoproteins, even though they did observe increase in HDL-c levels [36, 38].

These beneficial effects in the lipid profile resulting from Brazil nut consumption may be due to selenium’s ability to maintain the metabolically active form of thyroid hormones, as recent studies have shown that using thyroid hormone analogues such as eprotirome (KB2115) favors reduction in serum lipoproteins in euthyroid patients [6, 39].

Decreases in the non-atherogenic lipoprotein Apo A-1 levels observed in the current study are in line with previous studies in which patients displayed lower Apo A-1 levels after receiving thyromimetic medications [6, 39] and in hyperthyroidism [39]. On the other hand, we found no changes in the Apo B / Apo A-1 ratio, indicating no deleterious effect on reverse cholesterol transport, as a significant reduction of non-HDL cholesterol was observed.

Lp(a), one of the most atherogenic lipoproteins, is greatly determined (> than 90 %) by genetic factors and is little influenced by diet and lifestyle. Most lipid-lowering medications have no significant influence on Lp(a) [40]. It is well-established that many hormones have a strong effect on Lp(a) metabolism, and the thyroid hormone T_3_ seems to provide a significant reduction in plasma Lp(a) levels [5, 7]. Thus, the plasma Lp(a) level increases observed in the placebo group could be explained by the serum reduction of FT_3_ levels.

In the current study, a significant reduction in serum FT_3_ levels was observed only in the placebo group. These hormonal alterations could be explained by the effect of energy intake reduction, as previous studies have shown that these alterations can be diet-induced [41, 42]. On the other hand, the Brazil nut group maintained FT_3_ levels, which may be explained by the fact that selenium favors increases in the more metabolically active form of thyroid hormones [43–47].

The selenium status at baseline, in the sample population of the present study, was below the normal range of plasma selenium levels required to reach maximum glutathione peroxidase activity, established as 90 to 125 μg/l [18]. We suggest this occurs because of the increased oxidative stress in dyslipidemia [48].

The reduction in plasma selenium concentrations in the placebo group could be due to the nutritional recommendations for individuals with dyslipidemia, as the main food sources of this trace element are offal, seafood, meats, and cereals and grains [22]. The amount of selenium provided in this study (≈200 μg) can be obtained by consuming 03 units of Brazil nut (102 Kcal) daily.

The present study showed that a small restriction in energy intake might not influence an individual’s lipid profile; however, the consumption of Brazil nut in combination with the diet could significantly improve the serum lipids profile. Nevertheless, there were not found statistical differences in blood pressure and serum lipids between groups of intervention, possibly because the nutritional recommendation favored improves in these parameters in both groups and due the complexity of these patients with regard to disease duration, medication use and heterogeneity.

### Strengths and limitations

To our knowledge, this is the first randomized, double-blind, placebo-controlled study to investigate the effects of a personalized balanced diet associated with Brazil nut consumption in dyslipidemic and hypertensive patients. The present study has limitations that may have influenced the findings, including the group of participants who did not complete the study. Considering the potential public health implications of our results, there is a need for more randomized studies with larger patient groups, conducted in populations with a wider range of plasma selenium concentrations, and no medication use. In addition, *in vitro* studies are needed to evaluate the fractional T_3_ that would be affected by selenium supplements.

## Conclusion

The reduction in energy intake associated with the consumption of partially defatted Brazil nut flour (200 μg of selenium/day) contributed to a reduction in serum total cholesterol and non-HDL cholesterol levels in dyslipidemic and hypertensive patients undergoing drug treatment, without altering thyroid hormone concentrations.

## References

[1] Mendis S, Puska P, Norrving B, WHO (2011). Global atlas on cardiovascular disease prevention and control: policies, strategies, and interventions.

[2] Pande RL (2012). Approach to lipid therapy in the patient with atherosclerotic vascular disease. Curr Treat Options Cardiovasc Med.

[3] Berthezene F, Perrot L, De Parscau L, Valentin C, Richard L (1983). Thyroid hormones and the metabolism of lipoproteins. Ann Endocrinol (Paris).

[4] Sasaki S, Kawai K, Honjo Y, Nakamura H (2006). Thyroid hormones and lipid metabolism. Nihon Rinsho.

[5] Josseaume C, Lorcy Y (2008). Thyroid hormone analogs: an important biological supply and new therapeutic possibilities. Ann Endocrinol (Paris).

[6] Ladenson PW, Kristensen JD, Ridgway EC, Olsson AG, Carlsson B, Klein I (2010). Use of the thyroid hormone analogue eprotirome in statin-treated dyslipidemia. N Engl J Med.

[7] Grover GJ, Mellstrom K, Ye L, Malm J, Li YL, Bladh LG (2003). Selective thyroid hormone receptor-beta activation: a strategy for reduction of weight, cholesterol, and lipoprotein (a) with reduced cardiovascular liability. ProcNatlAcadSci U S A.

[8] Morkin E, Ladenson P, Goldman S, Adamson C (2004). Thyroid hormone analogs for treatment of hypercholesterolemia and heart failure: past, present and future prospects. J Mol Cell Cardiol.

[9] Park HT, Cho GJ, Ahn KH, Shin JH, Hong SC, Kim T (2009). Thyroid stimulating hormone is associated with metabolic syndrome in euthyroid postmenopausal women. Maturitas.

[10] Köhrle J, Jakob F, Contempré B, Dumont JE (2005). Selenium, thethyroid, and the endocrine system. Endocr Rev.

[11] Bianco AC, Salvatore D, Gereben B, Berry MJ, Larsen PR (2002). Biochemistry, cellular and molecular biology, and physiological roles of the iodothyronineselenodeiodinases. Endocr Rev.

[12] Schomburg L (2011). Selenium, selenoproteins and the thyroid gland: interactions in health and disease. Nat Rev Endocrinol.

[13] Navarro-Alarcón M, López-Garcia de la Serrana H, Pérez-Valero V, López-Martínez C (1999). Serum and urine selenium concentrations in patients with cardiovascular diseases and relationship to other nutritional indexes. Ann NutrMetab.

[14] Chang JC, Gutenmann WH, Reid CM, Lisk DJ (1995). Selenium content of Brazil nuts from two geographic locations in Brazil. Chemosphere.

[15] Chunhieng T, Pétritis K, Elfakir C, Brochier J, Goli T, Montet D (2004). Study of selenium distribution in the protein fractions of the Brazil nut. Bertholletiaexcelsa J Agric Food Chem.

[16] Jones DD, May KE, Geraci SA (2010). Subclinical thyroid disease. Am J Med.

[17] National Kidney Foundation/DOQI (2002). Clinical practice guidelines for chronic kidney disease: evaluation, classification, and stratification. Am J Kidney Dis.

[18] Millán Adame E, Florea D, Sáez Pérez L, Molina López J, López-González B, Pérez De La Cruz A (2012). Deficient selenium status of a healthy adult Spanish population. Nutr Hosp.

[19] World Health Organization (WHO) (1983). Guidelines for the conduct of tobacco smoking among health professionals. Report WHO.

[20] Gomes VB, Siqueira KS, Sichieri R (2001). Physical activity among a random sample of the Rio de Janeiro. Cad SaudePublica.

[21] Ainsworth BE, Haskell WL, Herrmann SD, Meckes N, Bassett DR, Tudor-Locke C (2011). Compendium of Physical Activities: a second update of codes and MET values. MedSci Sports Exerc.

[22] TACO (2011). Tabela Brasileira de Composição de alimentos.

[23] Agricultural Research Service. 2013. USDA National Nutrient Database for Standard Reference, Release 26. Nutrient Data Laboratory Home Page [http://www.ars.usda.gov/ba/bhnrc/ndl]

[24] Brazilian Cardiology Society (2007). IV Brazilian Guideline for Dyslipidemia and Prevention of Atherosclerosis, Department of Atherosclerosis of Brazilian Cardiology Society. ArqBrasEndocrinolMetabol.

[25] Brazilian Cardiology Society, Brazilian Society of Hypertension, Brazilian Nephrology Society (2010). IV Brazilian Guidelines on Hypertension. Arq Bras Cardiol.

[26] Wehmann RE, Rubenstein HA, Pugeat MM, Nisula BC (1983). Extended Clinical Utility of a Sensitive and Reliable Radioimmunoassay of Thyroid-Stimulating Hormone. South Med J.

[27] Ellis SM, Ekins RP, Pasternak CA (1975). The Radioimmunoassay of Serum Free Triiodothyronine and Thyroxine. Radioimmunoassay in Clinical Biochemistry.

[28] Fossati P, Prencipe L (1982). Serum triglycerides determined colorimetrically with an enzyme that produces hydrogen peroxide. Clin Chem.

[29] Allain CC, Poon LS, Chan CS, Richmond W, Fu PC (1974). Enzymatic determination of total serum cholesterol. Clin Chem.

[30] Warnick GR, NauckM RN (2001). Evolution of methods for measurement of HDL-cholesterol: from ultracentrifugation to homogeneous assays. Clin Chem.

[31] Frost PH, Havel RJ (1998). Rationale for use of non-high-density lipoprotein cholesterol rather than low-density lipoprotein cholesterol as a tool for lipoprotein cholesterol screening and assessment of risk and therapy. Am J Cardiol.

[32] Friedwald WT, Levy RI, Fredrickson DS (1972). Estimation of the concentration of low-density lipoprotein cholesterol in plasma, without use of the preparative ultracentrifuge. Clin Chem.

[33] Ledue TB, Collins MF, Ritchie RF (2002). Development of immunoturbidimetric assays for fourteen human serum proteins on the Hitachi 912. ClinChem Lab Med.

[34] Simó JM, Camps J, Gómez F, Ferré N, Joven J (2003). Evaluation of a fully-automated particle-enhanced turbidimetric immunoassay for the measurement of plasma lipoprotein(a). Population-based reference values in an area with low incidence of cardiovascular disease. Clin Biochem.

[35] Labat L, Dehon B, Lhermitte M (2003). Rapid and simple determination of selenium in blood serum by inductively coupled plasma-mass spectrometry (ICP-MS). Anal Bioanal Chem.

[36] Rayman MP, Stranges S, Griffin BA, Pastor-Barriuso R, Guallar E (2011). Effect of supplementation with high-selenium yeast on plasma lipids: a randomized trial. Ann Intern Med.

[37] Maranhão PA, Kraemer-Aguiar LG, de Oliveira CL, Kuschnir MC, Vieira YR, Souza MG (2011). Brazil nuts intake improves lipid profile, oxidative stress and microvascular function in obese adolescentes: a randomized controlled trial. NutrMetab (Lond).

[38] Cominetti C, de Bortoli MC, Garrido AB, Cozzolino SM (2012). Brazilian nut consumption improves selenium status and glutathione peroxidase activity and reduces atherogenic risk in obese women. Nutr Res.

[39] Berkenstam A, Kristensen J, Mellström K, Carlsson B, Malm J, Rehnmark S (2008). The thyroid hormone mimetic compound KB2115 lowers plasma LDL cholesterol and stimulates bile acid synthesis without cardiac effects in humans. ProcNatlAcadSci USA.

[40] Kostner KM, Kostner GM (2004). Factors affecting plasma lipoprotein(a) levels: role of hormones and other nongenetic factors. SeminVasc Med.

[41] Mathieson RA, Walberg JL, Gwazdauskas FC, Hinkle DE, Gregg JM (1986). The effect of varying carbohydrate content of a very-low-caloric diet on resting metabolic rate and thyroid hormones. Metabolism.

[42] Danforth E, Burger AG, Wimpfheimer C (1978). Nutritionally-induced alterations in thyroid hormone metabolism and thermogenesis. Experientia Suppl.

[43] Combs GF, Midthune DN, Patterson KY, Canfield WK, Hill AD, Levander OA (2009). Effects of selenomethionine supplementation on selenium status and thyroid hormone in healthy adults. Am J ClinNutr.

[44] Balázs C (2008). The effect of selenium therapy on autoimmune thyroiditis. OrvHetil.

[45] Thomson CD, McLachlan SK, Grant AM, Paterson E, Lillico AJ (2005). The effect of selenium on thyroid status in a population with marginal selenium and iodine status. Br J Nutr.

[46] Berger MM, Reymond MJ, Shenkin A, Rey F, Wardle C, Cayeux C (2001). Influence of selenium supplements on the post-traumatic alterations of the thyroid axis: a placebo-controlled trial. Intensive Care Med.

[47] Calomme MR, Vanderpas JB, François B, Van Caillie-Bertrand M, Herchuelz A, Vanovervelt N (1995). Thyroid function parameters during a selenium repletion/depletion study in phenylketonuric subjects. Experientia.

[48] Ferroni P, Basili S, Davi G (2003). Platelet activation, inflammatory mediators and hypercholesterolemia. CurrVascPharmacol.

